# The Effect of Arbutin on The Expression of Tumor Suppressor P53, BAX/BCL-2 Ratio and Oxidative Stress Induced by Tert-Butyl Hydroperoxide in Fibroblast and LNcap Cell Lines

**DOI:** 10.22074/cellj.2021.6902

**Published:** 2020-04-22

**Authors:** Shima Ebadollahi, Mahdi Pouramir, Ebrahim Zabihi, Monireh Golpour, Mohse Aghajanpour-Mir

**Affiliations:** 1. Department of Clinical Biochemistry, Faculty of Medicine, Babol University of Medical Sciences, Babol, Iran; 2. Student Research Committee, Babol University of Medical Sciences, Babol, Iran; 3. Cellular and Molecular Biology Research Center, Health Research Institute, Babol University of Medical Sciences, Babol, Iran; 4. Cellular and Molecular Biology Research Center, Student Research Committee, School of Medicine, Mazandaran University of Medical Sciences, Sari, Iran; 5. Department of Medical Genetics, School of Medicine, Tehran University of Medical Sciences, Tehran, Iran; 6. Department of Genetics, Faculty of Medicine, Babol University of Medical Sciences, Babol, Iran

**Keywords:** Arbutin, Fibroblast, LNCaP, Oxidative Stress, Tert-Butyl Hydroperoxide

## Abstract

**Objective:**

Arbutin (p-hydroxyphenyl-β-D-glucopyranoside) possesses beneficial functions including antioxidant, anti- inflammatory, and anti-tumoral activities. Due to the important role of oxidative stress and apoptosis in the successful treatment of cancer, understanding mechanisms that lead to apoptosis in cancer cells, is essential. The purpose of the current study was to evaluate the effect of arbutin on tert-butyl hydroperoxide (t-BHP)-induced oxidative stress and the related mechanisms in fibroblast and Lymph Node Carcinoma of the Prostate (LNCaP) cells.

**Materials and Methods:**

In this experimental study, the LNCaP and fibroblast cell lines were pre-treated with arbutin (50, 250 and 1000 μM). After 24 hours, t-BHP (30 and 35 μM) was added to the cells. Viability was measured (at 24 and 48 hours) using MTT assay. The antioxidant effect of arbutin was measured by FRAP assay. The mRNA expression of P53 and BAX/BCL-2 ratio were measured using quantitative polymerase chain reaction (PCR). The percentage of apoptotic or necrotic cells was determined using a double staining annexin V fluorescein isothiocyanate (FITC) apoptosis detection kit.

**Results:**

Arbutin pre-treatment increased the total antioxidative power and cell viability in the MTT assay and reduced BAX/BCL-2 ratio, P53 mRNA expression and necrosis in fibroblasts exposed to the oxidative agent (P<0.001). In addition, our results showed that arbutin can decrease cell viability, induce apoptosis and increase BAX/BCL-2 ratio in LNCaP cells at some specific concentrations (P<0.001).

**Conclusion:**

Arbutin as a potential functional β-D-glucopyranoside has strong ability to selectively protect fibroblasts against t-BHP-induced cell damage and induce apoptosis in LNCaP cells.

## Introduction

Oxidative stress is defined as disequilibrium between production and disposal of reactive oxygen species (ROS) (1). Free radicals and oxidant species can impose irreversible oxidative damage on a variety of indispensable cellular constituents including proteins, lipids, and nucleic acids (2). Oxidative stress causes the dysregulation of oncogenes and tumor suppressor genes such as *P53*. Excessive accumulation of ROS above the homeostatic threshold, is detrimental to cells and disturbs physiological mechanisms related to proliferation, apoptosis, angiogenesis, etc. (3).

Oxidative stress has a prominent role in the pathogenesis of different diseases, such as inflammatory diseases, diabetes, cardiovascular diseases, certain cancers, and neurodegenerative diseases (4). ROS induce DNA damage, genome variability, and cell proliferation. Arbutin (*p*-hydroxyphenyl-β-D-glucopyranoside) extracted from bearberry leaf (*Arctostaphyllos uva-ursi*) possesses various beneficial features (5, 6). Arbutin is broadly utilized as a cosmetic skin whitening agent due to its strong inhibitory effects on hydroxylation of tyrosine in melanin production pathway (7). Alongside its antiseptic, antibacterial, and diuretic features, *in vitro* studies have proven its anti-inflammatory, antioxidant, and antitumoral activities (8). The tumor suppressor gene *P53*, the most prevalent mutated gene found in 50% of human cancers, is identified as a genome protector that maintains genome stability. *P53* is mutated through a broad diversity of cellular insults, including DNA damage, oncogene activation, hypoxia, oxidative stress, and DNA-damaging chemotherapy agents (9). *P53* can induce genes such as pro-apoptotic genes (*e.g. Bax, Caspase-3, Apaf-1, *and* P53*-inducible gene) that causes deletion of cells through the incitement of cell mortality or senescence, and inhibit the aggregation of damaged cells (10, 11). The antiapoptotic mitochondrial protein Bcl-2 and the pro-apoptotic protein Bax are known to be vital regulators of programmed cell death (11). The* BAX/BCL-2* ratio as an index of the mitochondrial apoptotic pathway can control cytochrome c release from mitochondria to cell cytoplasm (12). Tert-butyl hydroperoxide (t-BHP), as a peroxide and an appropriate substitute for H_2_O_2_, is commonly utilized to investigate several cellular injuries such as oxidative-induced injuries, cell apoptosis, and the fundamental molecular mechanisms which are triggered by ROS (13). To widen the knowledge on the biological effects of arbutin, we investigated the effects of arbutin under oxidative stress conditions induced by t-BHP and evaluated its effects on the expression of tumor suppressor *P53* and the *BAX/BCL-2* ratio which are essential genes involved in programmed cell death.

## Materials and Methods

### Chemicals and reagents

In this experimental study, Dulbecco’s Modified Eagle Medium (DMEM) high glucose and RPMI-1640 were purchased from Biowest (Austria). Fetal bovine serum (FBS) and penicillin- streptomycin were bought from Gibco (Germany). Pure (98%) arbutin powder, 2, 4, 6-tripyridyls- triazine (TPTZ), and 3- [4, 5-dimethylthiazol-2-yl]-2, 5-diphenyltetrazolium (MTT) were purchased from Sigma-Aldrich (Germany). Annexin V-FITC apoptosis detection kit was purchased from eBioscience (San Diego, CA, USA). Tert-butyl hydroperoxide (t-BHP) was obtained from MERK (Germany) and cDNA synthesis Kit and YTA qPCR probe MasterMix 2x, were purchased from Yekta Tajhiz (Iran).

### Cell lines pretreatment and exposure

The fibroblast cell line was isolated from human newborn foreskin according to Pandamooz et al. (14) method, with the parents' informed consent and upon approval from the local Ethics Committee (Babol University of Medical Sciences, Babol, Iran) and the AR-positive human prostate cancer (PCa) LNCaP cell line was obtained from National Cell Bank of Iran (Pasteur Institute). The fibroblast and LNCaP cells were respectively cultured in DMEM high glucose and RPMI-1640, including 10% FBS, 100 IU/mL penicillin, and 100 μg/mL streptomycin. They were kept at 37˚C in a humidified atmosphere containing 95% air and 5% CO_2_. In all tests, cells were permitted to habituate for 24 hours before any treatments.

### Arbutin and t-BHP treatment

Oxidative stress was induced by introducing t-BHP into the culture media. The fibroblasts (10^4^ cells/well) and LNCaP (7×10^3^ cells/well) were cultured in 96- well plates. After 24 hours (60% confluency), the supernatant was replaced with three nontoxic concentrations of pure (98%) arbutin powder in complete medium (50, 250, and 1000 μM) for an additional 24 hours. To evaluate the t-BHP effects, 30 and 35 μM t-BHP were added to the wells containing arbutin in complete medium in fibroblast and LNCaP cells, respectively. The cells without arbutin and t-BHP were considered the control groups. Finally, after 24 and 48 hours of exposure to t-BHP, the supernatant was collected to perform FRAP assays, and the cells were washed twice with phosphate-buffered saline (PBS, pH=7.4) to measure cells viability using MTT assay.

### Measuring cell viability using MTT assay

Tetrazolium dye 3- [4, 5-dimethylthiazol-2-yl]-2, 5-diphenyltetrazolium bromide (MTT) is usually used to assess cells viability. The MTT-colorimetric assay is based on the capacity of viable cells to reduce MTT into formazan dye through succinate dehydrogenase in mitochondria. After exposure of the cells to arbutin with/ without consequent exposure to t-BHP and incubating for 24 and 48 hours, 50 μL of 5 mg/ml MTT in PBS was added to each well and incubated for another 4 hours. Afterward, the media were aspirated, and the formazan precipitate was dissolved in 150 μl dimethyl sulfoxide (DMSO) to lyse the cells. The color intensity of the solution was measured by Camspec-M501 spectrophotometer (Camspec, UK) at 570 nm with 630 nm as the reference wavelength. The results were reported as the percentage of the control ones (13).

### Estimation of ferric reducing antioxidant power

The Ferric Reducing Antioxidant Power (FRAP) assay was done according to Benzie and Strain (15) method. The FRAP assay evaluates the capacity of reduction of total ‘‘antioxidants’’ which are capable of reducing ‘‘Fe+3 2, 4, 6-tripyridyl-s-triazine (TPTZ) complex’’ to the bluecolored ferrous form at low pH. The assay mixture is made by adding same volumes of each sample (collected media at t=24 and 48 hours) and standards (50μl each) in 1.5 ml of FRAP reagent including 10 mM TPTZ in 40 mM hydrochloride acid, 0.3 mM acetate buffer (pH=3.6), and ferric chloride 20 mM. The absorbance was measured (after 15 minutes incubation at 37˚C) at 593 nm of wavelength. Standard graphs were constructed using different concentrations of FeSO_4_ (125- 1000μM) (16).

### Quantitative reverse transcription polymerase chain reaction assay

Total RNA was extracted from treated cells For quantitative reverse transcription polymerase chain reaction (qRT-PCR), using RNA extraction mini kit (Yekta Tajhiz, Iran) according to the manufacturer’s instructions. cDNA synthesis kit was utilized to synthesize the cDNA library. The reaction mixture included 1 μl of the random hexamer, 10 μl of RNA, and 2.4 μl of diethyl pyrocarbonate (DEPC)-treated H_2_O. After gentle mixing and brief centrifuging, the mixture was incubated at 70˚C for 5 minutes. Then, while chilling on ice, 4 μl of 5X loading buffer, 1 μl of Moloney Murine Leukemia Virus (MMLV) Reverse Transcriptase, 1 μl dNTPs, and 0.5 μl RNasin were added, and the mixture was incubated for 60 minutes at 37˚C, then heated at 70˚C for 5 minutes. For *Bax*, *Bcl-2*, and *GAPDH* detection, mRNA PCR primers were designed by Primer 3 software and synthesized by Pishgam company (Iran). Primer sequence homology and total gene specificity were determined by BLAST analysis (http://www.ncbi.nlm. nih.gov/blast) (Table 1).

**Table 1:** List of primer sequences used for quantification of mRNA expression


Genes	Primer sequence (5ˊ-3ˊ)

Bax	F: GGTTGTCGCCCTTTTCTACTTTGC
	R: ATGTCCAGCCCATGATGGTTCTG
Bcl-2	F: ATGTGTGTGGAGAGCGTCAAC
	R: AGCCAGGAGAAATCAAACAGAGG
GAPDH	F: GGTGGTCTCCTCTGACTTCA
	R: GTTGCTGTAGCCAAATTCGT


Subsequently, 100 ng of cDNA was used as the template in a qRT-PCR reaction using the YTA Super SYBR® Green qPCR Master Mix 2x (Yekta Tajhiz, Iran) kit, according to the manufacturer’s instructions. The reaction mixture, including 10μl of 2 X master mix, 0.4μl of forward primer, 0.4μl of reverse primer, 1μl of cDNA, 7.8μl of ddH_2_O and 0.4μl of passive reference dye. The PCR thermal cycling situations were set as follows: 40 cycles of denaturation at 95˚C for 10 seconds, annealing at 60˚C for 10 seconds, extension at 72˚C for 20 seconds and a final extension at 72˚C for 7 minutes. For evaluation of *P53* expression, 100 ng of cDNA was used as the template in a qRT-PCR reaction using a TaqMan *TP53* primer and probe was purchased from Applied Biosystems (Foster City, CA, USA). The *TP53* sequence (Assay ID Hs01034249_m1) was amplified in a 20μl reaction containing 10μl of qPCR probe Master Mix 2x, 2μl of cDNA, 1μl of a TaqMan P53 Gene (primers and probes), and 7μl of DNase-free water. PCR cycling steps were as follows: 3 minutes at 94˚C, 40 cycles of 15 seconds at 95˚C, and 1 minute at 60˚C. A TaqMan GAPDH (Applied Biosystems, FosterCity, CA, USA, Assay ID Hs03929097-g1) was used as a reference gene (17). The expression level of *P53* and *Bax*, *Bcl-2* genes was evaluated by qRT-PCR using an ABI 7500 Fast Real-Time PCR System (Applied Biosystems). In order to analyze the expression of related genes, we used the formula 2^-∆∆CT^ in which ∆∆*CT*=∆*CT* sample-∆*CT* reference for calculating the fold expression of each transcript relative to *GAPDH*, as a housekeeping gene.

### Annexin V-fluorescein isothiocyanate/propidium iodide apoptosis assay

LNCaP and fibroblast cells were cultured in six-well plates (25×10^4^ cells/well) for 24 hours and then pretreated with different concentrations of arbutin (50, 250 and 1000 μM) for 24 hours followed by exposure to t-BHP (30, and 35 μM) for extra 24 and 48 hours. Apoptosis was investigated by an annexin V-FITC apoptosis detection kit based on the manufacturer’s instructions. After washing the cells twice with cold PBS, cells were collected and centrifuged at 1500 rpm for 5 minutes at 4˚C. Then, they were resuspended in 1 ml binding buffer. The cells were incubated with annexin V-FITC for 5 minutes and then incubated with propidium iodide for 15 minutes in the dark at room temperature 25˚C finally, the percentages of apoptosis and necrosis were observed using FACS Calibur flow cytometer (BD Biosciences, San Jose, CA, USA).

### Statistical analysis

All the data obtained under normal and oxidative stress conditions, are presented as mean ± standard error of three separately performed experiment. One-way ANOVA with post-hoc test (Tukey) was used for statistical comparison, and P<0.05 were contemplated statistically significant (0.01<*P<0.05, 0.001<**P<0.01, * ** P<0.001).

## Results

### Dose-response relationship of arbutin and t-BHP toxicity

**Fig.1:**
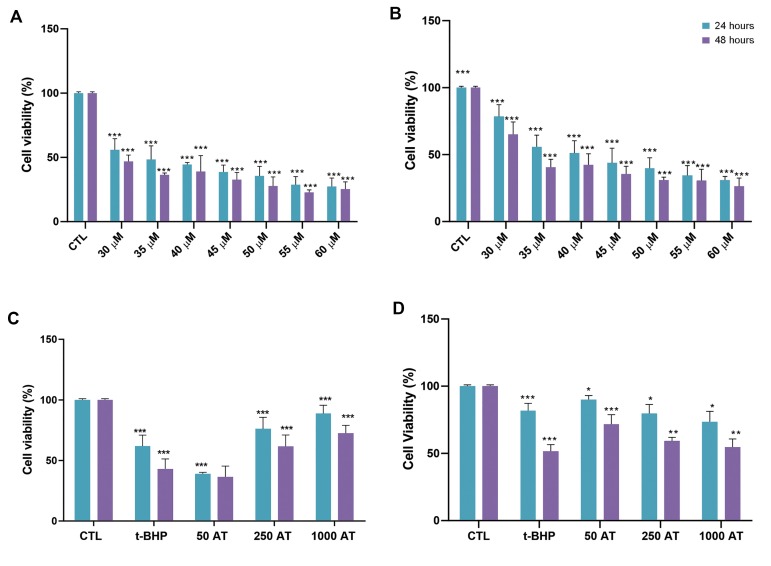
The protective effects of arbutin on t-BHP-induced cytotoxicity in fibroblast and LNCaP cells. **A.** The t-BHP toxicity in fibroblast and **B.** LNCaP cells (*** ; P <0.001 versus control). **C. **The Effect of arbutin pre-treatment on fibroblast and **D.** LNCaP cells viability after 24 and 48 hours of exposure to t-BPH. Data shown represent the mean values of three experiments ± SD (*; P<0.05, **; P<0.01, ***; P<0.001 as compared to oxidant group). CTL; Control group, t-BHP; Tert-butyl hydroperoxide, 50 AT+t-BHP; Arbutin 50 μM with 30 μM t-BHP, 250 AT; Arbutin 250 μM with 30 μM t-BHP, and 1000 AT; Arbutin 1000 μM with 30 μM t-BHP.

We first assessed the dose response relationship for t-BHP, a potent pro-oxidant, in fibroblast (Fig.1A) and LNCaP cells (Fig.1B). Toxic effects in fibroblast and LNCaP cells and viability were evaluated after 24 and 48 hours of exposure to varying concentrations of t-BHP, using MTT assay. The viability of the cells significantly reduced after incubation with t-BHP in a dose-dependent manner (30-60 μM, P<0.001). The 30 and 35 μM of t-BHP were used for further experiments to determine the effect of arbutin in fibroblast and LNCaP cells, respectively. Moreover, we evaluated the toxicity of arbutin after 24 and 48 hours of exposure. The MTT assay showed that arbutin decreased cell viability at doses above 1000 μM. Then, we used three nontoxic doses (50, 250, 1000 μM) of arbutin for further experiments.

### The effect of arbutin pre- treatment on the oxidative stress induced by t-BHP in fibroblast and LNCaP cell lines

Pre-treatment with 250 and 1000 μM arbutin after 24 and 48 hours of exposure to t-BHP, significantly increased cell viability compared to the oxidant group exposed only to 30 and 35 μM t-BHP alone in fibroblasts (Fig.1C) and LNCaP cell lines, respectively (P<0.001, Fig.1D).

### The effect of arbutin on ferric reducing antioxidant power in fibroblasts and LNCaP cell lines

**Fig.2:**
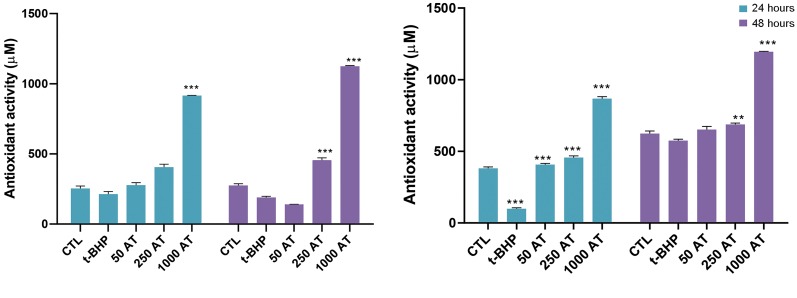
Effect of arbutin on total antioxidant capacity. The ferric reducing antioxidant power (FRAP) after pre-incubation with arbutin in** A.** t-BHP-induced fibroblast and **B.** LNCaP cells. Fibroblast and LNCaP cells were pre-treated with arbutin (50, 250 and 1000 μM) and exposed to t-BHP (30 μM) for 24 and 48 hours. CTL; Control group, t-BHP; Tert-butyl hydroperoxide, 50 AT+t-BHP; Arbutin 50 μM with t-BHP 30 μM, 250 AT; Arbutin 250 μM with t-BHP 30 μM, 1000 AT; Arbutin 1000 μM with t-BHP 30 μM, ** ; P<0.01, and * ** ; P<0.001 versus tBHP.

We found that following treatment of the fibroblast and LNCaP cells with tBHP at 30 and 35 μM for 24 hours, FRAP decreased in the supernatant of the cells compared to the control groups (P<0.01, n=3). Also, after 24 and 48 hours of pre-treatment of cells with arbutin 250 and 1000 μM, the antioxidant power increased markedly in the supernatant of fibroblast (Fig.2A) and LNCaP (Fig.2B) cells in t-BHP-induced oxidative stress.

### Effect of arbutin pre-treatment on *BAX/ BCL-2* ratio and *P53* mRNA expression in t-BHP-induced oxidative stress

**Fig.3:**
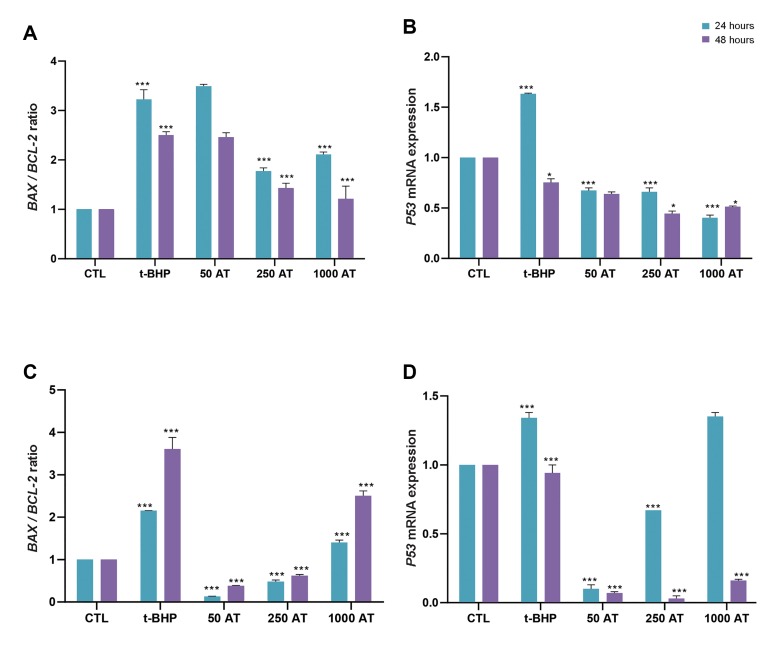
Effect of arbutin on *BAX/BCL-2* ratio and *P53* mRNA expression.The *BAX/BCL-2* ratio and *P53* mRNA expression in **A, B.** t-BHP-induced fibroblast and **C, D.** LNCaP cells. CTL; Control group, t-BHP; Tert-butyl hydroperoxide, 50 AT+t-BHP; Arbutin 50 μM with t-BHP 30 μM, 250 AT; Arbutin 250 μM with t-BHP 30 μM, and 1000 AT; Arbutin 1000 μM with t-BHP 30 μM (0.01<*P<0.05, 0.001<**P<0.01, and *** P<0.001 versus tBHP).

The *BAX/BCL-2* ratio (Fig.3A) and *P53* mRNA expression (Fig.3B) was considerably increased after 24 hours of exposure to t-BHP (30 μM) in fibroblasts compared to the control group (P<0.001). Expression of *P53* mRNA in fibroblasts after 24 and 48 hours of pre-treatment with arbutin (50, 250 and 1000 μM) and 30 μM t-BHP, is illustrated in Figure 3B. Pre-treatment with arbutin (250 and 1000 μM) after 24 and 48 hours of exposure to t-BHP, significantly reduced *BAX/BCL- 2* level (Fig.3A) and *P53* mRNA (Fig.3B) compared to the oxidant group only exposed to 30 μM t-BHP (P<0.001). Moreover, the ratio of *BAX/BCL-2* mRNA expression was considerably increased after 24 and 48 hours exposure to t-BHP (35 μM) in LNCap cells in comparison to the control group (P<0.05, Fig.3C). As illustrated in Figure 3C, in LNCap cell line, pretreatment with arbutin (50, 250 and 1000 μM) could significantly decrease the *BAX/BCL-2* ratio compared to the group exposed t-BHP (35 μM, P<0.05). Also, after 48 hours of pre-treatment with 1000 μM arbutin, *BAX/BCL- 2 *ratio markedly increased compared to the control group in LNCaP cells (P<0.001). Expression of *P53* mRNA increased after 24 hours of exposure to t-BHP compared to the control group in LNCaP cells and pre-treatment with arbutin 50 and 250 μM significantly decreased *P53* mRNA expression compared to both control and oxidant groups (P<0.05, Fig.3D). Moreover, after 48 hours of pre-treatment with arbutin (50, 250 and 1000 μM), *P53* mRNA expression significantly diminished compared to both control and oxidant groups (P<0.05, Fig.3D).

### Effect of arbutin pre-treatment on t-BHP induced apoptosis and necrosis in LNCaP and fibroblasts

**Fig.4:**
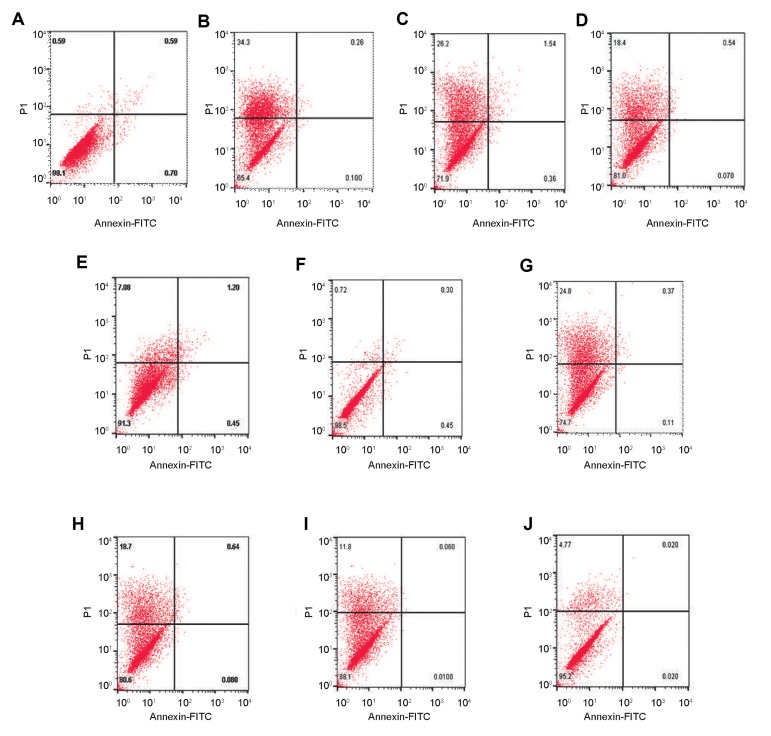
Effect of arbutin on the t-BHP-induced cytotoxicity in fibroblast cells. Arbutin pre-treatment inhibited necrosis of human fibroblast cells in a dosedependent manner after **A-E.** 24 hours and** F-J.** 48 hours exposure to t-BHP. The necrosis rate of cells cultured in the **A, F. **Control, **B, G.** 30 μM tert-butyl hydroperoxide, **C, H.** 50 μM arbutin+30 μM t-BHP, **D, I.** 250 μM arbutin+30 μM t-BHP, and **E, J.** 1000 μM arbutin+30 μM t-BHP

In fibroblasts, exposure to t-BHP increased the necrosis rate from 0.59% (Fig.4A) to 34.3% (Fig.4B) after 24 hours. The pre-treatment with 50, 250 and 1000 μM arbutin decreased necrosis induced by t-BHP after 24 hours, from 34.3% (Fig.4B) to 26.2% (Fig.4C), 18.4% (Fig.4D) and 7.08% (Fig.4E).

Additionally, after 48 hours exposure to t-BHP increased the necrosis rate from 0.72% (Fig.4F) to 24.8%(Fig.4G). The pre-treatment with 50, 250 and 1000 μM arbutin arbutin decreased necrosis induced by t-BHP in fibroblast cells from 24.8% (Fig.4G) to 18.7% (Fig.4H), 11.8% (Fig.4I) and 4.77% (Fig.4J).

**Fig.5:**
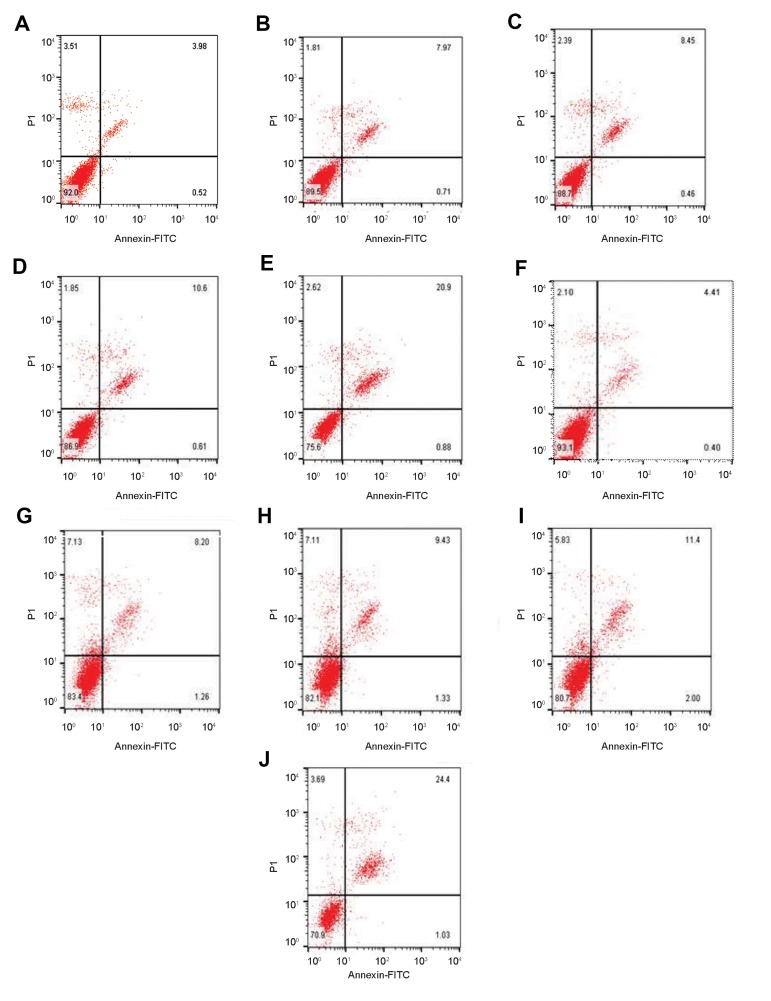
Effect of arbutin on t-BHP- induced cytotoxicity in LNCaP cells. Arbutin induces apoptosis in human LNCaP cells in a dose-dependent manner after **A-E.** 24 hours and **F-J.** 48 hours exposure to t-BHP. The apoptosis rate of cells cultured after 24 hours exposure to t-BHP in the **A, F.** Control, **B, G.** 35 μM tert-butyl hydroperoxide, **C, H.** 50 μM arbutin+35 μM t-BHP and **D, I.** 250 μM arbutin+35 μM t-BHP, and **E, J.** 1000 μM arbutin+35 μM t-BHP.

To assess whether arbutin-induced cytotoxicity is indeed due to induction of apoptosis, rather than necrosis in cells, we performed flow cytometry analysis using Annexin V-FITC/ PI double-staining method. Conspicuously, LNCap cells exposure to arbutin resulted in enhanced late apoptosis in a dose-dependent manner. As shown in Figure 5, LNCaP cells exposure to t-BHP increased the apoptosis rate from 4.50% (Fig.5A) to 8.68% (Fig.5B) after 24 hours. Also, pre-treatment with 50, 250 and 1000 μM arbutin after 24 hour increased the apoptosis rate to 8.91% (Fig.5C), 11.21% (Fig.5D) and 21.78% (Fig.5E). As illustrated in Figure 5F, t-BHP promoted apoptosis from 4.81% (Fig.5F) to 9.46% (Fig.5G) compared to the control group. Moreover, pre-treatment with 50, 250 and 1000 μM arbutin after 48 hours, increased the percentage of apoptotic cells induced by t-BHP from 9.46% (Fig.5G) to 10.76% (Fig.5H), 13.4% (Fig.5I) and 25.43% (Fig.5J) respectively

## Discussion

PCa is the most common solid tumor and the sixth main reason for cancer deaths among men, worldwide. It is currently considered one of the foremost important medical issues that the male population faces (18, 19). There has been an enormous interest in using natural agents capable of prompting programmed cell death in cancer cells, which can develop the mechanism-based prevention and treatment approaches for cancer (20). As far as we are concerned, the effect of arbutin has not been evaluated against t-BHPinduced cytotoxicity in LNCaP and fibroblast cells. Besides antiseptic, skin whitening, anti-inflammatory and anti-tussive properties of arbutin, it might have the potential to be an anti-tumor and anti-oxidative agent which could be related to *P53* regulation (8, 21). Tert-butyl hydroperoxide as a potent oxidative stress stimulator has been used to induce oxidative damage *in vitro* and *in vivo* (13). In this experiment, the effect of arbutin was evaluated in LNCaP and fibroblast cells in t-BHP-induced oxidative stress. Due to the vital role of programmed cell death in successful cancer treatment, it is precious to understand the mechanisms that trigger apoptosis, especially *P53*-mediated apoptosis in cancer cells (22). Since apoptotic cell pathways are regulated by the expression level of specific genes, especially the *BAX/BCL-2* ratio, evaluation of the *BAX/BCL-2* ratio can determine the apoptotic pattern in the cells (23).

Results of the current study showed that arbutin decreased *BAX/BCL-2* ratio, and *P53* mRNA expression, increased cell viability and total antioxidant capacity in fibroblast cells and led to diminished t-BHP-induced cell death. Moreover, arbutin induced apoptosis, increased *BAX/BCL-2* ratio, and reduced cell viability in LNCaP cell. There are many documents which illustrated that natural compounds decrease intracellular ROS and protect cells from oxidative stress. It was reported that Turkish propolis rich in phenolic as well as flavonoid contents, significantly decreased t-BHP induced oxidative stress in human fibroblast cells. Moreover, quercetin and rutin protected Caco-2 cells and L6 myoblasts from t-BHP induced oxidative stress (24). It was reported that arbutin in combination with ursolic acid, can act as a strong UVprotector in fibroblast cell (25). However, so far, no study reported the cytoprotective effect of arbutin in fibroblast cells exposed to t-BHP. The protective effect of arbutin (250 and 1000 μM) was illustrated by the substantial increase in fibroblasts viability and FRAP level under stressed conditions (30 μM t-BHP).

On the contrary, in our experiment, arbutin (50 μM) decreased fibroblast cells viability to levels even lower than the t-BHP group. It may be because arbutin at this dose could not resist the oxidant situation and changed to a pro-oxidant substance. Previous studies reported that natural antioxidants like flavonoids and polyphenols, can act as a pro-oxidant when they are exposed to alkali pH, oxygen, and high concentration of transition metals (26). Some antioxidants (resveratrol, coumaric acid, and N-acetylcysteine) could act as pro-oxidant, increased ROS production and led to cell damage in the endothelial cells (27). These investigations raised the possibility that arbutin might have anti-cancer activities for instance against prostate tumor cells. Inconsistent with our data, *in vitro* and* in vivo* experiments confirmed that arbutin induced free radical-scavenging, anti-hyperglycemic, antioxidant, and anti-inflammatory effects and could enhance the level of FRAP in the supernatant of different cells (28-30). Also, pre-treatment of the retinal ganglion cells (RGCs) cells with arbutin (100 μM) had protective effects against oxidative damage induced by H_2_O_2_ (31). The results of our study declared that pre-treatment with arbutin downregulated *BAX/BCL-2* ratio and *P53* mRNA expression in fibroblast cells compared to the oxidant group. The results support previous reports concerning cytoprotective and antioxidant features of arbutin obtained *in vitro* and *in vivo* (8).

Our findings are consistent with the results showing antioxidative effects of arbutin as a potent radical scavenger, in isolated human neutrophils, murine microglial BV2, and Hep G2 cell lines (28, 32, 33). Also, arbutin can reduce oxidative stress derived from the melanogenic pathway within the skin (34). According to previous studies, P53 was significantly up-regulated in an oxidative stress situation and could cause cell cycle arrest, cellular senescence, and apoptosis (35).

Interestingly, we observed a decrease in necrosis and *P53* mRNA expression in fibroblasts in response to arbutin pre-treatment in t-BHP-induced oxidative stress groups. It was shown that arbutin declines radical hydroxyl production and protects U937 cells from *Bax*mitochondrial pathway apoptosis (36). Our analysis of annexin-v/PI, flow-cytometric results revealed that pretreatment with 250 μM and 1000 μM of arbutin, increases apoptosis in LNCaP cells exposed to t-BHP (35 μM). Small polyphenols, such as gallic acid, and quercetin, can exhibit peroxidation activity (37). We found that t-BHP treatment increases *BAX/BCL-2* mRNA ratio and pre-treatment with arbutin may counteract *t*-BHPinduced upregulation of BAX/BCL-2 ratio, however, in comparison to the control group, suggesting that arbutin may trigger t-BHP-induced apoptosis in LNCaP cell in a dose-dependent manner. Our results are in consistency with the results of a previous study done on the inhibitory properties of arbutin on the proliferation of cancer cells, including A375 human malignant melanoma cells through up-regulating *P53* expression (38), as well as, HCT-15 and TCCSUP cells (39). Moreover, Jiang et al. reported that arbutin and its acetylated derivative significantly reduce cell viability, promote cell apoptosis, decrease the expression of *Bcl-2* and *Bcl-xL*, and induce a mitochondrial disruption in B16 murine melanoma cells. Treatment with arbutin was shown to induce caspase 9, 3, and PARP, increase *BAX/BCL-2* ratio in cells and cause DNA damage by mitochondrial apoptotic pathway (40). Moreover, the results of this study in terms of *BAX/BCL 2* ratio and apoptosis indicated a more intense effect for arbutin in extended periods. According to flow cytometry results, the rate of late apoptosis was higher than early apoptosis in LNCaP cell, which probably reveals the effect of arbutin on DNA damage, and cell membrane changes. This may reflect that arbutin, in addition to its effect on the cell membrane, may disrupt cell cycle. It seems that arbutin is a potent agent to be used against LNCaP cells. The anticancer feature of natural polyphenols is generally attributable to their various pharmacological effects such as anti-inflammatory, anti-oxidative, and anti-proliferation effects. They modulate PCa cell growth by modulating molecular events, and signaling cascades associated with cell survival, proliferation, migration, and differentiation, immune responses, angiogenesis, hormone activities, etc. (18).

Our findings confirmed that arbutin acts as an antioxidant agent, and has anti-proliferative activity in LNCaP cells via induction of apoptosis. Moreover, arbutin caused favorable changes within the fibroblasts, thereby protecting them from oxidative stress conditions. More studies are required to investigate the combined effects of arbutin and chemotherapeutic agents in prostate cancer.

## Conclusion

This study indicated, for the first time, that arbutin can increase total antioxidant power leading to significant protective effects on fibroblasts against t-BHP-induced oxidative stress. Also, results of this study revealed that arbutin, which does not show significant toxicity at concentrations up to 1000 μM, could serve as a potential candidate with strong protective effects on t-BHPinduced oxidative stress, by increasing cell viability and decreasing necrosis in fibroblasts. Also, arbutin (1000 μM) can induce apoptosis and increase *BAX/BCL-2* ratio in LNCaP cell line in t-BHP-induced oxidative stress. These findings provide basis for further investigations on arbutin as a novel therapeutic agent to combat oxidative stress for treatment of various diseases.
